# Investigations of ripple pattern formation on Germanium surfaces using 100-keV Ar^+^ ions

**DOI:** 10.1186/s11671-015-0751-4

**Published:** 2015-02-28

**Authors:** Indra Sulania, Dinesh Agarwal, Mushahid Husain, Devesh Kumar Avasthi

**Affiliations:** Inter University Accelerator Centre, Aruna Asaf Ali Marg, New Delhi, 110067 India; Jamia Millia Islamia, Jamia Nagar, New Delhi, 110025 India

**Keywords:** 07.79.Lh, 81.16.Rf, 79.60-I, 61.72.uf, 79.60-i, Germanium, Ion bombardment, Atomic force microscopy, Sputtering

## Abstract

We have investigated the formation of nanoripples on the surface of germanium, Ge(100), due to the effect of 100-keV Ar^**+**^ ion irradiation. The irradiation was carried out at different incidence angles from 0° to 75° in steps of 15° with respect to the surface normal with a fixed ion fluence of approximately 3 × 10^17^ ions/cm^2^. Atomic force micrographs show an increase in surface roughness from 0.5 to 4.3 nm for the pristine sample and the sample irradiated at 60° incidence angle due to cos^−1^(*θ*) dependence on sputtering yield. With increase in angle of incidence, there is transition observed from nanodots to aligned nanodots perpendicular to the direction of the beam. There is an increase in size of the nanostructures observed from 44 to 103 nm with angle of incidence. The formation of nanoripples initiates at an angle of *θ* ~ 45°. Ripple pattern formation has taken place on the Ge surface in the energy regime of 100 keV as compared to the other reports which had been carried out using very low energy ions. Raman spectra reveal that the near surface of crystalline Ge samples becomes amorphous due to interaction of Ar^+^ ions due to creation of defects through collision cascades.

## Review

### Introduction

Ion beam is a unique tool used for creating nanopatterns at the surfaces of a desired substrate. Earlier low-energy ion irradiation techniques had been used extensively for etching purposes to clean materials’ surfaces for device applications. As an outcome of the etching process, formation of various surface patterns such as ripples or dots or pits has been observed by many research groups [1-4]. The ordering of these surface patterns depends upon the ion beam parameters such as time of bombardment, energy of the ions used, and angle of incidence of the ion beam. With proper control of ion beam parameters and proper understanding of the physics of ion-solid interactions and defect dynamics, fabrication of surface nanostructures can be achieved in a desired manner [5,6]. As a consequence of low-energy ion irradiation, the near-surface region of the target material gets amorphized due to creation of point defects through the process of collision cascades. Collision cascade or displacement cascade is the sequential collisions of atoms, which is induced due to the incoming energetic particle within a solid target. If the energy of the incoming ion in a collision cascade is higher than the threshold displacement energy of the atom in a material, the atom will be displaced from its lattice site with production of point defects such as vacancy or interstitial. Having passed the surface plane, the incident ion will undergo further collisions generating secondary recoils or cascades. These collision cascades are statistical in nature and follow a Gaussian-like depth distribution, *f*(*x*)d*x*, thus leading to the formation of nanostructures on the surface of a material through the process of self-assembly [5,7-10]. The formation of ripples due to Ar^+^ ion bombardment was observed by Navez et al. on the surface of glass during the etching process [11]. These ripples have the same analogy as the ripples which occur naturally on sand dunes in deserts and the river beds or sea water due to the flow of air. The first efforts to explain this experimentally observed phenomenon were put by Bradley and Harper [12]. They proposed a linear theory based upon Sigmund’s theory of sputtering [13]. According to the linear theory, the main mechanism governing such changes on the surface is the competition or interplay between the roughening induced by ion bombardment and smoothening due to surface diffusion. It could explain many of the experimental findings but did not explain the saturation phenomenon of ripple amplitude and formation of dots or pits on the surfaces as observed by many of the experimental groups on various target materials. The understanding of these features could be achieved only by adding non-linear terms in the linear theory, and thus, non-linear theories came into the picture which could explain most of the experimental findings [14,15].

Germanium (Ge) is being chosen over Si-based devices due to its high carrier mobility which shows improved results in metal-oxide-semiconductor field-effect transistors (MOSFET) technology when used with high-*k* dielectric materials [16-18]. The Si/Ge-based heterostructure is being studied by many research groups for its usefulness in photocurrent applications, absorption, photoluminescence spectroscopy, etc. [19]. Carbone et al. bombarded Ge(001) with 1-keV Xe^+^ ions in a GISAXS setup in ESRF at Grenoble, France. They observed the early stage of ripple formation on Ge(100) surfaces under near-normal ion beam sputtering with a wavelength of 42 nm [20]. Yehuda et al. studied the dynamics of defect creation in amorphous Ge thin films with a thickness of 200 to 300 nm using Ar^+^ ions in the energy range of 15 to 110 eV at 30° incidence [21]. They observed formation of voids in the films, with the films remaining amorphous in nature. It is further concluded that the incident ion energy does not affect the formation of defects but rather ion fluence plays a crucial role in defect creation. Ziberi et al. observed the formation of well-ordered nanodots and nanoripples on Si and Ge surfaces during low-energy ion beam erosion with a mean wavelength of approximately 45 nm upon bombarding it with different ions and variable energies from 0.5- to 1.5-keV ion energies. They also observed an increase in wavelength with increases in ion energies [22,23]. Chason et al. studied the surface roughening of Ge(001) due to 200-eV Xe^+^ ion bombardment during the molecular beam epitaxy to see the surface diffusivity of Ge on Ge(100) [24]. It was found that the initially smooth surfaces reach a steady state roughness which depends on the temperature and incident ion adatom flux. Sulania et al. observed the formation of an amorphized Ge layer on crystalline Ge(100) due to the impact of 1.5-keV Ar atoms incident at 0° with respect to the surface normal [25]. It is concluded that the nanostructure formation does take place on Ge but the structures are not well organized and non-uniform in size. The size of the structures and rms roughness increase with increases in ion fluence. Teichmann et al. studied the ripple coarsening behavior on the surfaces of Ge, Si, Al_2_O_3_, and SiO_2_ using ions with energies of 600 and 1,200 eV at ion incidence angles of 65° and 75°. The ion fluence was varied from 1.1 × 10^17^ to 1.3 × 10^19^ ions/cm^2^. They observed the coarsening process to be independent of the material used [26]. Zhou et al. studied the formation of ripple using a focused ion beam (FIB)-scanning electron microscope setup using 30-keV Ga^+^ on the Ge(001) surface [27]. It is concluded that the ripple direction changes with the change in fast scan direction of FIB. As the dose of Ga^+^ is increased to 8.67 × 10^15^ ions/cm^2^, nanostructured networks are formed on the surface of Ge(001). In another work using FIB on GaAs, Xu et al. found ordered droplets of Ga on the surface of GaAs and observed an increase in size with ion fluence [28]. Wu et al. observed the formation of self-organized nanoripples on thin films of SrTiO_3_ deposited on STO under the influence of FIB irradiation at different ion beam currents with a characteristic wavelength of approximately 375 nm and amplitude of approximately 100 nm [29].

All the above studies on Ge had been carried out at a very low energy regime of the ions or electrons (0.07 to 1.5 keV) with the main motivation of studying the defect formation on Ge with low-energy ion irradiation. In few of the reports, FIB is used to form the ordered structures on the surfaces of various materials to ensure the concentrated impact of the ion beam onto the surface to form structures.

The range of the low-energy Ar ion of few keVs is approximately 200 Å inside Ge. The electronic and nuclear energy losses of 1.5-keV Ar are 4.3 and 33.5 eV/Å, respectively. On the other hand, the range of 100-keV Ar ion inside Ge is approximately 722 Å, and the electronic and nuclear energy losses are 36.5 and 59.6 eV/Å, respectively (as estimated by SRIM 2003) [30]. Clearly, the dominating process is the nuclear energy through which the energy is deposited within the system and the changes in surface morphology occur due to collision cascade. Therefore, in this paper, we report the formation of Ge nanoripples due to the impact of medium-range energy ions of 100 keV. Moreover, the angle of incidence variation on Ge has not been studied in detail for such a large range starting from 0° to 75° incidence with respect to the surface normal. Therefore, in this study, we investigate the formation of surface nanostructuring on the Ge(100) surface due to the effect of change in incidence angle of the 100-keV Ar^+^ ion beam.

### Experimental details

We have used 100-keV Ar^**+**^ ions to bombard the samples of Sb-doped Ge(100) at 300 K. The angle of incidence of the ion beam with respect to the surface normal has been changed from *θ*_ion_ = 0°, 15°, 30°, 45°, 60°, and 75° keeping the other parameters constant. The base pressure of the chamber during bombardment was around 2 × 10^−6^ mbar. An ion fluence of 3 × 10^17^ ions/cm^2^ was used (corresponding to a bombardment time of approximately 27 min at a flux of 18.5 μA/cm^2^). The ion fluence was chosen from the study on fluence dependence on Ge using 500-keV Ar^4**+**^ ion energy (not reported here). Below this fluence, the surface patterning was not observed but only roughness was visible on the surface of Ge(100) as observed in our previous experiment with medium-range energy ions. The angle variation was obtained by changing the angle of the ladder on which samples were mounted by rotating it. The experiment was performed in the material science beam line of the Low Energy Ion Beam (LEIB) facility of IUAC, New Delhi. We analyzed the results obtained with the help of atomic force microscopy (AFM) micrographs by observing the change in shape, size, and roughness variation of the structures obtained on the surface of Ge. The surfaces of pristine and bombarded samples were investigated by using AFM Nanoscope IIIa Multimode from Digital Instruments, Canberra, USA. All measurements were conducted in air using silicon-nitride (RTES) tips from Bruker (Singapore) with a nominal tip radius of <10 nm. The micro-Raman measurements were performed on the pristine and irradiated samples using a Renishaw inVia micro-Raman setup (Renishaw, Gloucestershire, UK) using an Ar ion laser with an excitation wavelength of 514.5 nm. The AFM and Raman measurements were performed at the Inter University Accelerator Centre (IUAC), New Delhi.

### Results

**Figure 1:**
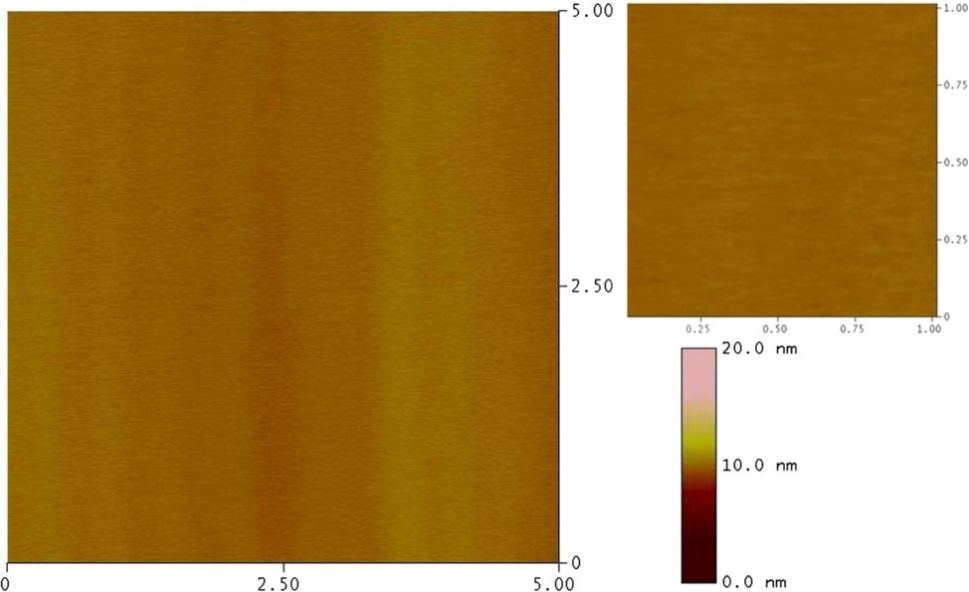
**AFM micrographs of pristine Ge(100) sample in 5 × 5 μm** ^**2**^ **and 1 × 1 μm** ^**2**^ **scan sizes.**

**Figure 2:**
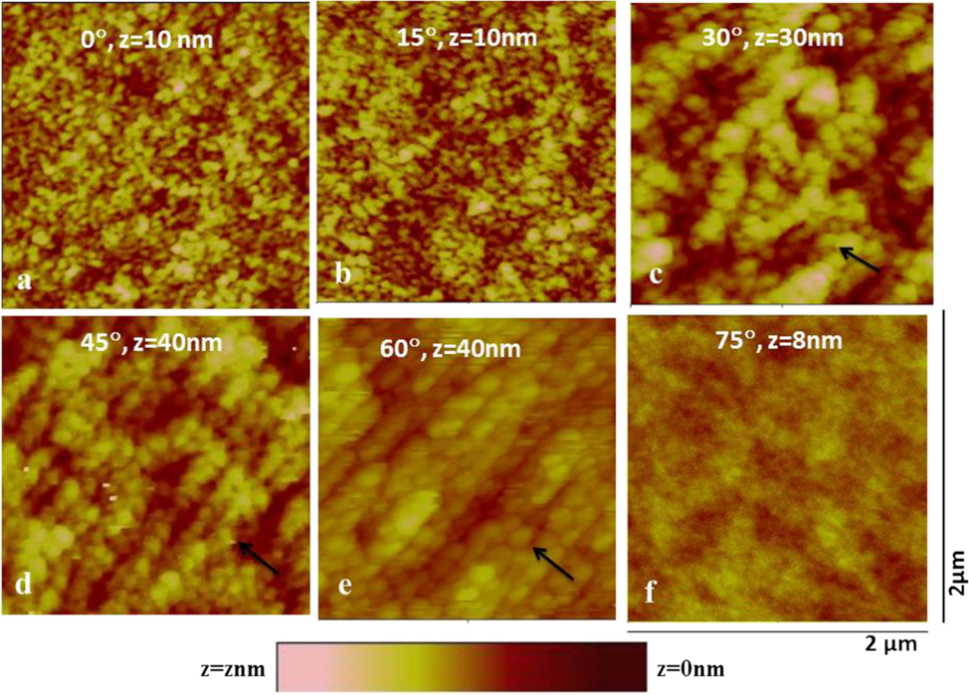
**AFM micrographs of the irradiated at fluence 3 × 10** ^**17**^ **ions/cm** ^**2**^ **sample of Ge(100).** At angles of incidence of **(a)** 0°, **(b)** 15°, **(c)** 30°, **(d)** 45°, **(e)** 60°, and **(f)** 75° (all images in 2 × 2 μm^2^ scan size).

**Figure 3:**
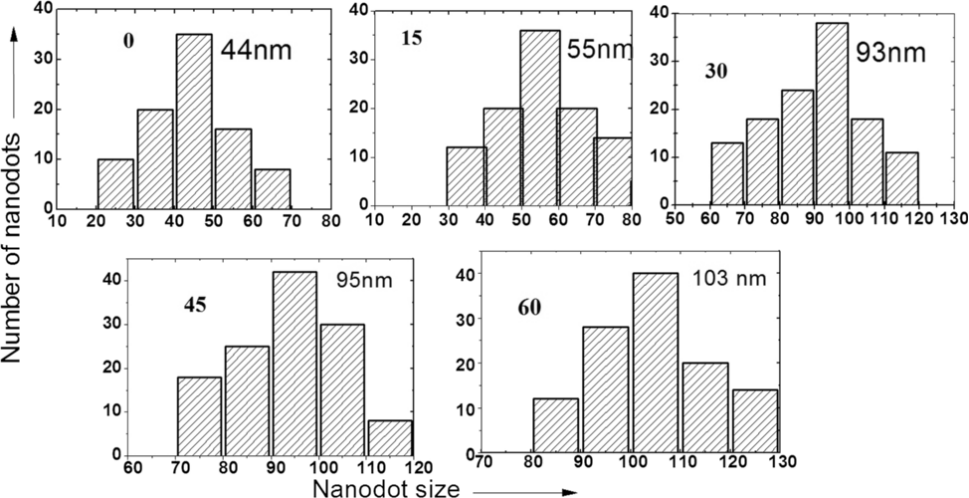
**Size distribution of the nanodots for different angles of incidence.**

The AFM micrograph of the pristine Ge(100) sample is shown in Figure 1. The surface of the pristine sample is smooth with a rms roughness of 0.2 nm. Figure 2 shows the surface morphology of the samples irradiated at different angles of incidence with respect to the surface normal using 100-keV Ar^+^ ions with a fluence of approximately 3 × 10^17^ ions/cm^2^. It is clear from the AFM micrographs that when the sample was irradiated at 0°, the dot structures started to evolve on the surface of Ge(100) with initial increases in rms roughness to 0.5 nm as compared to 0.2 nm of the pristine sample. The average size of the nanodots was found to be approximately 44 nm as determined by the line profile of the AFM image (Figure 3). At 15° incidence, the dot size increases to 55 nm and the roughness to 0.97 nm. On further increasing the angle to 30°, the alignment of the dots was observed perpendicular to the direction of the beam (shown with the arrow). The rms roughness increases to 3.4 nm, and the size of the dots increases to 93 nm. The wavelength of the aligned pattern was observed to be 120 nm. At 45°, the alignment becomes prominently visible perpendicular to the beam direction. This is the transition angle for ripple pattern to form on Ge. The rms roughness increased to 3.7 nm. The size of the dots was observed to be 95 nm and wavelength of the aligned nanodots as 128 nm. On further increasing the angle to 60°, the roughness of the samples increases to 4.3 nm. The alignment was more prominent and the formation of ripples at this angle is well observed, although the ripples are not continuous in nature. The wavelength of the ripples was found to be 140 nm and size of the dots observed to be 103 nm (Figure 3). Bradley et al. have shown that for the single element material, the formation of continuous or ordered ripples is less probable as compared to the one with binary material [31]. For the case of InP bombarded with 1.5-keV Ar ions, we have observed the formation of ripple pattern at 63° angle of incidence [4]. Moreover, the ripples were found growing continuous on the surface of the sample unlike those seen as chains of dots on Ge in the present case. The direction of the ripple formation was along the beam direction in InP [4] unlike Ge where the direction of ripples is perpendicular to the direction of the beam (shown by the arrow in Figure 2). Due to the single element, the surface sputtering takes place uniformly on the material, thus giving rise to chains of dots pattern. At an incidence angle of 75°, it was observed that the surface looks similar to that of the pristine sample, with slight increase in the roughness value of 0.3 nm as compared to 0.2 nm of the pristine sample. At this angle, the reflection of ions takes place which decreases the probability of interaction of the ions with the sample surface and does not change much in comparison to the pristine sample.

**Figure 4:**
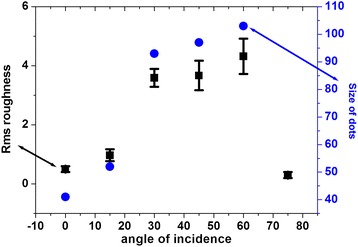
**Graph between angle of incidence of ion beam with rms roughness and size of nanodots.**

The variation in rms roughness and size of dots with angle of incidence of the ion beam is plotted in Figure 4. The size of the dots and rms roughness keep on increasing with the angle of incidence and reaches the maximum value for 60° and again decreases to 0.3 nm for the 75° incidence angle. This happens because as the angle is changing, the ion beam is penetrating in lesser depths and hence the sputtering would be more from the surface of Ge. Therefore, the roughness is found to be maximum for 60°. The nanopatterns appear on the surface of a material due to the interplay of surface diffusion and sputtering due to the impact of energetic ions. This is minimum at 75° incidence angle due to lesser ion interaction with the surface atoms and also a decrease in ion-induced diffusion.

**Figure 5:**
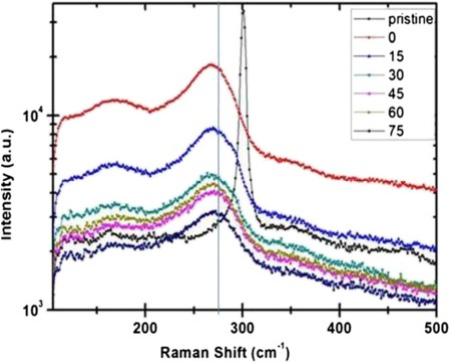
**Raman spectra of the pristine and irradiated Ge samples at different angles.**

It is well reported in literature that low-energy ion beam irradiation leads to amorphization of surfaces due to creation of point defects through collision cascades, and further irradiation induces the restructuring of the amorphous layer in the form of nanostructures such as dots or ripples depending on the angle of incidence of the ion beam [32]. The thickness of the amorphous layer greatly depends upon the angle of incidence of the ion beam which is qualitatively studied by Raman measurements. Raman spectra of the pristine and irradiated samples are shown in Figure 5. The sharp peak which corresponds to crystalline Ge is visible at 302.5 cm^−1^ for the pristine sample. The amorphous Ge peaks start to evolve around 268.8 cm^−1^ for the 0° incidence, and the crystalline peak vanishes as the sample was irradiated with a fluence of 3 × 10^17^ ions/cm^2^. The peak is asymmetric in nature. When the irradiation angles were increased further, a little shoulder is observed at approximately 290 cm^−1^ which may correspond to the nc-Ge peak [33]. The intensity of the shoulder peak decreases monotonically with increase in angle of incidence of the ion beam. This might be due to the change in the thickness of the amorphous layer which is scaling with the intensity of the Raman peak in Figure 5.

### Discussion

Nanopattern formation takes place on the surface due to the interplay between the surface diffusion term which could be ion induced or thermal diffusion on the surface or a combination of both and sputtering of the material from the surface due to the impact of energetic ions onto the surface. According to the BH theory, the ripple formation takes place on the surface due to the difference in the sputtering rates at different surface curvatures. The troughs are eroded faster as compared to the crests [12]. In the case of a binary compound, the difference in the sputtering rates also plays a significant role in the pattern formation onto a surface due to different masses present inside the material. The lighter element will sputter more readily than the one with higher mass, as seen by different research groups for InP [4], GaAs [34], Gasb [5], etc. But for the single element material, it may be difficult to achieve the ripple formation, although there are contradictory reports on this especially for the cases of Si targets [2,10] but not so clearly visible on Ge [18]. We have observed ripple pattern formation on the Ge surface; however, the structures are not continuous but are chains of nanodots, and there was continuous ripple formation on InP. This also supports the theory proposed by Bradley et al. in their PRL report [31] which suggested that ripples readily form on a binary compound rather than on a single element material. The rms roughness increases with increases in incidence angles with cos^−1^(*θ*) dependence on sputtering yield. Further surface roughness is maximum for 60° and minimum for 75° incidences, respectively.

**Figure 6:**
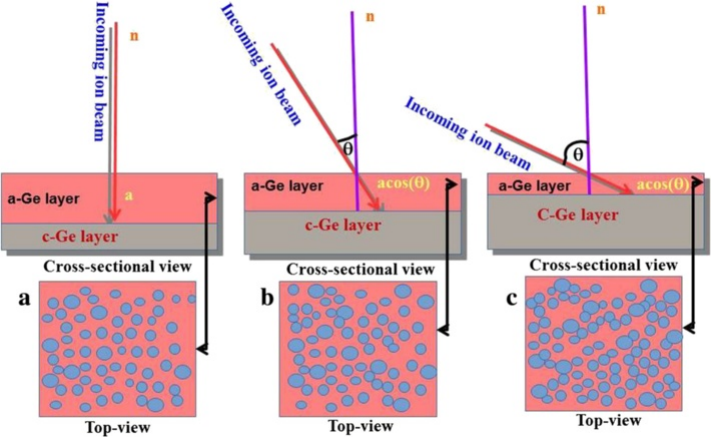
**Schematic representation of ion beam falling at different angles with respect to surface normal onto Ge surface. (a)** 0°, **(b)** 30°, and **(c)** 60°.

The Raman analysis shows that as the angle of incidences was increased, the thickness of the amorphous layer decreases due to lesser penetration depth of the Ar^+^ ion inside Ge. The amorphous Ge peak does not shift with change in angle of incidence which indicates that no strain is developed in the samples upon irradiation. Since, the range of 100-keV Ar^**+**^ ions inside Ge is approximately 72 nm [30] and the penetration depth of the Ar ion laser with a wavelength of 514 nm is about 20 nm [35,36], the crystalline Ge peak is not observed for the case of irradiated samples. The schematic representation of the ion beam penetrating the Ge sample at three different incidence angles is shown in Figure 6. The incident beam strikes the sample surface at an angle (*θ*) with respect to the surface normal. In particular, it is shown that three samples are irradiated with the same Ar^+^ ion fluence or dose but at different incidence angles (0°, 30°, and 60°) with respect to the surface normal. Figure 6 shows the top views of the corresponding schematic and represents the formation of nanostructures on the surface. The thickness of the amorphous layer is higher in the case of normal incidence irradiation as shown in Figure 6a because of the higher projected ion range for 0° as compared to higher angle. At normal ion beam irradiation, the dots are formed in an irregular symmetry due to the random strike of ions on the sample surface, and as the angle of irradiation is increased, the alignment of dots has been observed in the direction perpendicular to the beam direction (Figure 6b) which is more pronounced for the case of higher angle as visible in Figure 6c. As the angle of irradiation is increased, the thickness of the amorphous layer decreases which is scaled with the intensity of the Raman peak in Figure 5. Decrement in relative intensity of the a-Ge peak in Raman clearly indicates the lesser thickness of the amorphous layer at higher angles as represented by the schematic in Figure 6. The Raman analysis qualitatively depicted the reduction in thickness of the amorphous layer with increase in ion beam irradiation angle, as we are only getting the signal from the amorphous or modified layer of the Ge surface due to ion irradiation which becomes lesser for higher incidence angles. The signal from the bulk or unmodified Ge is missing in the irradiated samples due to less penetration of the 514-nm Ar laser used in the Raman characterization.

## Conclusions

The formation of nanostructures is observed on Ge by changing the incidence angle of the ion beam. The nanodots are observed for normal incidence angle, and these nanodots are aligned perpendicular to the direction of the beam as the incidence angle is increased. The surface roughness of the irradiated samples is found to increase with increase in angle of incidence from 0.3 to 4.6 nm. The size of the nanostructures increases from 44 to 103 nm, and the ripple wavelength increases from 97 to 140 nm with angle of incidence. The micro-Raman analysis revealed that the Ge is getting amorphized upon irradiation due to interaction of the Ar^+^ ion with Ge atoms, creating defects through collision cascades.
